# An attachment-based pilot program to promote adolescent adjustment to parental divorce

**DOI:** 10.1186/s13034-024-00729-9

**Published:** 2024-03-30

**Authors:** Karla Tay-Karapas, Mónica Guzmán-González, Fabiola Gómez, Priscila Comino, Karmele Salaberria, Joaquín Bahamondes

**Affiliations:** 1 Escuela de Psicología, Facultad de Psicología, Departamento de Psicología Clínica y de la Salud y Metodología de Investigación, Karla Tay-Karapas, Universidad Católica del Norte, Universidad del País Vasco, Avenida Angamos 0610, Antofagasta, San Sebastián, España Chile; 2 Escuela de Psicología, Mónica Guzmán-González, Universidad Católica del Norte, Antofagasta, Chile; 3 Fabiola Gómez, Facultad de Psicología, Universidad de Talca , Talca , Chile; 4 Departamento de Psicología Evolutiva y de la Educación, Facultad de Educación y Deporte, Universidad del País Vasco, Vitoria-Gasteiz, España; 5 Karmele Salaberria, Facultad de Psicología, Departamento de Psicología Clínica y de la Salud y Metodología de Investigación, Universidad del País Vasco, San Sebastián, España; 6 Joaquín Bahamondes, Escuela de Psicología, Universidad Católica del Norte, Antofagasta, Chile

**Keywords:** Parental divorce, Parental attachment, Life satisfaction, Positive affect, Negative affect, Intervention, Adolescents

## Abstract

The study aimed to assess the impact of an attachment-based intervention on adolescent adaptation to parental divorce. The Adolescent Adjustment Pilot Program to Parental Divorce (AAPPD) employed an experimental group format, targeting improvements in various adaptation indicators (life satisfaction, positive affect, and negative affect). The sample comprised 30 Chilean adolescents aged 12 to 16 (*M* = 13.6, *SD* = 1.35), with 60% females and 40% males. After the intervention, the adolescents showed a decrease in negative affect at 6 and 12 months. However, no differences were identified in other dimensions of subjective well-being considered as indicators of divorce adaptation. The findings prompt discussion on theoretical and clinical implications.

## Introduction

Although adolescence involves the pursuit of autonomy, the emotional well-being of adolescents is strongly influenced by their environment and aspects related to their family [1, 2]. One significant family process that can occur in the lives of children and adolescents is divorce, which can have long-term effects on their well-being and mental health [3–6]. Studies in this field have reported that children of divorced parents often experience lower life satisfaction levels than those from intact families [7–9].

Moreover, divorce is considered a process that can have implications for attachment relationships between parents and children [3, 6]. Research conducted in the context of divorce shows that children of divorced parents report less secure attachment bonds compared to children of married parents [9, 10]. Additionally, in a recent study with adolescents, children of separated parents were found to associate subjective well-being with the quality of attachment with their mothers [11].

Despite this background and the accumulated knowledge in understanding the effects of divorce on children, there is a scarcity of studies focused on the design and implementation of intervention strategies to promote the adaptation of adolescents going through parental divorce. Within the limited available evidence on divorce interventions, it is primarily focused on the school stage and addressing internalizing or externalizing symptoms once they have already manifested [3, 12–16]. However, developing interventions that foster children’s adaptation to divorce is particularly relevant in this critical stage of the life cycle, aiming to contribute to healthy socioemotional development [5]. This study seeks to contribute in this area through the implementation of an intervention aimed at promoting adaptation to divorce in adolescents themselves, from an attachment-based approach.

## Attachment and adaptation to divorce

The parental separation or divorce, terms used in the research as analogous concepts to describe the cessation of cohabitation between parents [17], constitutes a process that unfolds over time, typically beginning years before the divorce and extending years after the legal dissolution [5]. Divorce can be conceived as a stress-generating transition that involves the restructuring of the family system [3, 12, 18]. While there is no consensus definition regarding adaptation to divorce, some common elements observed in various conceptualizations suggest that it refers to the process by which an individual or a family adjusts to the changes and challenges resulting from the dissolution of a marriage [17, 18]. Among such challenges are the ability to cope with the emotional, social, and economic stress associated with divorce, as well as the ability to establish new routines, roles, and family relationships in a healthy and functional manner [18].

In the case of adolescents, the existence of a specific definition of adaptation to divorce is unknown; however, literature on the subject typically considers subjective well-being as an indicator of adaptation. Subjective well-being is a multidimensional construct that refers to how individuals evaluate and experience their lives, considering both positive and negative aspects [19, 20]. It consists of three main elements: a cognitive dimension, referring to life satisfaction, and two aspects related to the affective dimension: positive and negative affect [21]. Life satisfaction involves the cognitive evaluation that a person makes about their own life [19], and it can manifest in two ways: the overall assessment of life or in specific areas such as family, school, and friendships, among others [22]. On the other hand, positive and negative affect constitute the emotional component of subjective well-being, reflecting a person’s emotional state at a given moment [23]. Positive affect is associated with the experience of positive emotions such as happiness, fulfillment, vitality, and tranquility, while negative affect is linked to unpleasant moods such as anger, sadness, and fear [24].

Furthermore, divorce constitutes a significant event capable of altering the attachment bonds that adolescents have with their parents [3, 6]. Attachment theory posits that emotional bonds established in childhood with significant figures, in this case, parents, influence the formation of relationship patterns and coping abilities throughout life [25]. The attachment bond between a child and their primary caregiver undergoes changes throughout all stages of life [25]. A parent-child relationship based on trust, open communication, and the absence of alienation plays a crucial role in forming a secure bond [26]. These aspects are linked to traditional parental elements such as warmth and communicative interaction [27]. From the quality of these early interactions, Internal Working Models (IWM) are constructed, reflecting the perception that children develop about their own worthiness of receiving love and attention, as well as the availability of others to effectively meet their needs [28]. These IWMs will influence the mental representations that adolescents or adults later use to understand the environment around them, as well as multiple aspects of individual and relational functioning [25, 29, 30]. In this regard, there is ample evidence that the quality of attachment bonds is associated cross-sectionally and longitudinally with dimensions of subjective well-being throughout the life cycle [1, 31–34]. Therefore, considering previous evidence indicating decreases in well-being levels in adolescents whose parents have divorced, a possible intervention approach to prevent adaptation problems to this experience is to strengthen the attachment bond between adolescents and their parents.

## Interventions in divorce

The majority of available studies on divorce interventions focus on separating partners [35–38] or on post-divorce co-parenting and shared parenting [39–45]. To a lesser extent, there is evidence regarding specific interventions with children of divorced parents that have shown positive empirical results [46–49]. However, there is a lack of studies that have intervened with adolescents who are children of divorced parents.

From another perspective, research has demonstrated the effectiveness of attachment-based interventions during adolescence [50–52]. This approach is further supported by a recent meta-analytic analysis that identified significant effects of small to moderate magnitude for attachment-based interventions, which remained evident up to six months after the intervention [53].

The review of these background studies reveals that both divorce interventions and attachment-based approaches have primarily focused on working with parents rather than directly involving adolescents [14, 53]. This highlights a significant gap in research related to interventions that center on the direct experience of adolescents, providing them with support to navigate this family transition. Adolescents are often considered more independent and capable of handling their emotions, which can lead to a lack of attention to their specific experience. The parental divorce process can have a significant impact on the emotional, social, and academic development of adolescents [5, 54–58]. Additionally, the inherent changes in the developmental stage, characterized by modifications in cognitive, socio-affective, behavioral, and moral domains, further contribute to the challenges [59]. Therefore, the development of interventions to help adolescents navigate this process in a healthy manner is necessary. Exclusively focusing on interventions with parents, with the assumption that this will benefit adolescents, fails to capture the complexity of their challenges and interactions. Instead, redirecting research toward an approach that includes adolescents themselves can foster self-reflection, emotional self-regulation, and the strengthening of their bonds with parents and others in general. In this way, it can contribute to their well-being and their ability to face life challenges, particularly those arising from their parents’ divorce.

## The present study

Based on the aforementioned background, the present study aimed to evaluate the effect of an attachment-based pilot intervention designed to promote adaptation to divorce in adolescents over time. The Adolescent Adjustment Pilot Program to Parental Divorce (AAPPD) adopts an experimental design and is structured as a group intervention with organized activities that incorporate attachment work towards both parents. The goal is to achieve improvements in divorce adaptation, specifically in levels of life satisfaction, as well as positive and negative affect in participating adolescents.

Our research expands prior knowledge in several ways. Firstly, this is the first study to our knowledge that assesses the impact of an intervention to promote divorce adaptation in adolescents, placing them as protagonists and differentiating itself from the adult-centric perspective that has prevailed in research. Secondly, this research incorporates attachment theory into its design, providing a solid conceptual framework that has inspired the development of multiple effective interventions. Thirdly, in terms of methodology, four measurements at different time points are included, increasing the possibility of assessing changes and their stability over time.

As a hypothesis, we expect that participants assigned to the intervention group will exhibit better levels of divorce adaptation at 3 months post-intervention (higher life satisfaction and positive affect, and lower negative affect) compared to those assigned to the control group, and that these changes will persist at 6 and 12 months.

## Method

### Participants

The inclusion criteria for the sample were: being Chilean, aged between 12 and 16 years, enrolled in school,

whose parents has been separated less than 1 year ago, considering as the criteria the cessation of cohabitation between the parents. Exclusion criteria included: having a diagnosis of severe psychopathology, receiving psychiatric or psychological treatment in the last year, and having any special educational needs and/or intellectual disability.

Initially, a sample of 46 adolescents was accessed, of which 9 were excluded for not meeting one or more of the inclusion/exclusion criteria; 3 of them, despite having parental consent (signed consent form), chose not to participate; and another 4 adolescents, having initially agreed to participate and having been authorized by their parents, did not show up on the day when the pre-intervention assessments (pre-test) began, nor at the start of the intervention itself.

Based on the above, the final sample consisted of 30 Chilean adolescents aged between 12 and 16 years (*M* = 13.6, *SD* = 1.35), with 18 females (60%) and 12 males (40%). Regarding the developmental stage, 19 (63.3%) were in early adolescence, while 11 (36.6%) were in late adolescence. Regarding the type of educational institution they attended, 43.3% attended a public school, 30% attended a subsidized private school, and 26.6% attended a private school. As for the marital status of the parents, 100% were children of divorced parents. Participants were randomly assigned to two groups of 15 participants each (control group and experimental group), balanced by sex and age. The control group did not receive the intervention but was evaluated at four time points (pre-test and post-test: 3, 6, and 12 months); however, given that variables linked to mental health were evaluated, if any adolescent in the control group obtained risk results or expressed interest in being accompanied, the possibility of receiving psychotherapeutic support was granted. While the experimental group participated in the ten sessions that comprised the intervention and was also evaluated at four time points (pre-test and post-test: 3, 6, and 12 months).

### Design

Was employed a 3 × 2 mixed design with groups (control and experimental) as a between-subjects factor, post-intervention measures (3, 6, and 12 months) as a within-group factor, and divorce adjustment (life satisfaction, positive affect, and negative affect) as the dependent variable. Pre-intervention measures of life satisfaction, positive affect, and negative affect were entered as covariates to statistically control for their initial levels.

### Procedure

After obtaining approval from the ethics committee of the Universidad Católica del Norte, Chile, and the ratification of the ethics committee of the Universidad del País Vasco, Spain, contact was made with different educational establishments in the city of Antofagasta, Chile. Through these institutions, access to the families of the participating adolescents was facilitated. Subsequently, a meeting space was created with the parents. After confirming the inclusion and exclusion criteria mentioned in the previous section, information about the nature of the research, data management, and the voluntary nature of their participation was provided. Additionally, parents were briefed on some of the general activities to be carried out during the intervention, in which their participation and support from home would be required. Finally, each parent individually authorized their child’s participation in the study by signing an informed consent form.

Following this, a meeting was arranged with the adolescents. After obtaining their assent to participate, pre-test evaluation questionnaires were administered, and the Adolescent Adjustment Pilot Program to Parental Divorce (AAPPD) intervention was initiated. Subsequent follow-ups were conducted, and evaluation instruments were administered again at 3, 6, and 12 months after the intervention (post-test). Faced with the possibility that some of the adolescents scored at risk levels in the dimensions evaluated, there was an action protocol, which included: informing the adolescent and their parents, and offering referral for psychotherapeutic care at the psychosocial intervention and counseling center.

The AAPPD pilot intervention program is based on group intervention with adolescents and consists of 10 sessions of 120 min each, distributed weekly. It was created ad hoc specifically for this work and has not been implemented previously. The program focuses on promoting secure attachment between adolescents and their mothers and fathers and enhancing self-esteem. The intervention was implemented by a mixed pair of therapists, both with similar postgraduate training and specialization in child and adolescent mental health. Each week, participants gather at a community center that provides the ideal space in terms of privacy and equipment for group work with adolescents. Table 1 summarizes the pilot treatment/intervention program:

**Table 1:** Summary of the Adolescent Adaptation Pilot Program to Parental Divorce (AAPPD)

Session	Topic	Objective	Intervention Focus
1	Group Cohesion	Facilitating the development of a sense of belonging, connection, and group identity in adolescents.	Collaboration Emotional support Satisfaction Mutual commitment
2–4	Enhancement of Self-Esteem	Empowering adolescents to strengthen aspects related to their self-esteem.	Self-awareness Self-acceptance Self-care
5–9	Promoting Attachment with Mothers and Fathers	Fostering adolescents to develop more secure attachment bonds with their mothers and fathers.	Exploration of origins Quality of communication and trust with parents Conflict resolution Belonging-differentiation
10	Closure Process	Facilitating adolescents to experience a proper and satisfactory conclusion to the therapeutic journey shared by group members.	Skill reinforcement Reflection on the process (evaluation, feedback, and conclusions) Preparation for the future

During the first session, there was an initial introduction of each group member (therapist pair and participants), based on a set of questions included in an introductory activity. The goal of the first session is to assist adolescents in developing group cohesion, promoting an environment in which members feel connected, supported, and committed to each other. It is noteworthy that this is a cross-cutting objective that was reinforced throughout the entire intervention.

From the second to the fourth session, the focus is on strengthening aspects related to the self-esteem of adolescents, always considering the specific context of divorce that parents and children are going through. In the second session, elements of emotional, cognitive, bodily, and social self-awareness were addressed (for example, through exercises that involve the ability to recognize and understand one’s own thoughts, beliefs, emotions, sensations, and mental states). In the third session, the focus was on self-acceptance, helping them learn to embrace their entirety rather than focusing only on the negative parts (for example, a group activity in which each adolescent takes on the role of one of their peers, providing an external but close view of who they are and what they desire in life). In the fourth session, the emphasis is on self-care, which is promoted through knowledge and practice of actions that can be consciously and voluntarily performed to maintain, improve, or preserve their physical, mental, and emotional health (for example, planning collective self-care spaces with their mothers and/or fathers, separately). At the end, and given that a significant number of sessions have taken place, a brief evaluation and feedback on the experience are conducted.

Between the fifth and ninth sessions, the objective is to foster the development of secure attachment bonds between adolescents and their parents, considering the context of divorce. The fifth session begins with exploring origins: family structure, dynamics, and interactions, all tailored to the participants’ age and the context of group intervention. For example, at the end of the previous session, participants are asked to create a drawing of their family genogram for this session, accompanied by family photos for collaborative work (previously communicated to parents via email, allowing them to support the activity at home and ideally complete it together). The genogram serves as a tool to represent the structure of each family with its history, relationships, hierarchies, providing adolescents with a framework to understand their heritage, accompanied by photos that playfully connect their life development to their family history.

In the sixth and seventh sessions, the focus is on the quality of communication and trust between parents and children. Two sessions are allocated to these themes as they are central elements in attachment work and are often interconnected. One activity involves a series of games where adolescents identify situations in their interaction with parents, along with associated emotions or experiences. They then share these in a plenary session led by therapists, discussing communication, trust, and both positive and negative emotions experienced in their relationship with their parents. Another activity is the writing of letters between parents and children (supported from home by parents), where adolescents draft a letter expressing emotions and thoughts to their parents that they might find challenging to verbalize. They focus on positive aspects of the relationship, including elements of communication and trust they would like to improve with their parents. Parents are then asked to send a similar letter for their child before the next session. During the session, in an emotionally supportive environment, adolescents can read their letters privately in smaller workgroups, followed by reflections on communication and trust between parents and children, guided by therapists.

In the eighth session, the focus is on conflict resolution between parents and children. Using a series of illustrations containing behavioral and emotional expressions (primarily conflictual) between parents and children, adolescents are asked to express their thoughts on what they believe is happening in the scene, how they think the conflict was resolved, and what they have done to resolve situations where they had disagreements with their parents. Negotiation and respect for agreements are highlighted in the reflection. Another activity involves role-playing, where therapists take on the role of adolescents, and participants assume the role of mothers or fathers, acting out conflict situations with parents and providing the respective resolution. This allows adolescents to empathize with their parents’ role in their upbringing.

The ninth session focuses on working on belonging-differentiation as a process that adolescents go through to balance their needs to establish their individual identity while also seeking a sense of belonging to their family or community. This is considered with the specific characteristics of families going through divorce. For example, an activity titled “From Dependency to Belonging” was conducted. Adolescents were invited to work in small groups to create a timeline through illustrations, depicting their journey from early childhood to adolescence, including the moment of their parents’ divorce. They were asked to mention situations within their families that made them feel dependent, autonomous, alone, accompanied, etc. Subsequently, all timelines were presented in a plenary session, and a debate was initiated where therapists modeled the synthesis of the activity by creating a timeline that captured the main ideas and represented the group. Emphasis was placed on differences between dependence and belonging, changes in their needs from childhood to adolescence, individuation, rights versus responsibilities and commitments, experiences associated with divorce in relation to the family, etc. At the end of the session, elements from previous meetings and the current one (communication, trust, conflict resolution, belonging-differentiation) were gathered, and the adolescents collectively created a decalogue with key ideas to promote healthy relationships between parents and adolescent children. This decalogue was transcribed and sent via email to each of the parents.

In the tenth and final session, the goal is for adolescents to experience a satisfactory closure of the process, celebrating progress, and preparing them to continue their personal journey with the tools and support they have acquired during the group intervention. For example, individual and collective skills are reinforced through group dynamics, and a mailbox where they left notes week by week reflecting on their experience in the space, their perception of the therapists who accompanied them, etc., is opened and discussed.

### Variables measured and instruments used

#### Life satisfaction

This variable was measured with the Life Satisfaction Scale (SWLS, Diener et al. [60], Spanish adaptation of Atienza et al. [61], adapted and validated in Chile for the juvenile population by Tay-Karapas & Yárnoz-Yaben [62]. The SWLS is a unidimensional measure that evaluates the level of satisfaction with life in a factor composed of 5 items (for example: “I am satisfied with my life”), to which is answered according to a scale of 5 points (1 totally disagree, 5 totally agree). Higher scores are indicative of higher levels of life satisfaction. For the present study the reliability index omega was 0.74.

#### Positive and negative affect

Positive affect (PA) and negative affect (NA) were measured with the Brief Scale to Evaluate Positive and Negative Affection (PNA-10, Yárnoz-Yaben et al. [63]), adapted and validated in Chile for the juvenile population by Tay-Karapas & Yárnoz-Yaben [64]. It is a 10 item scale, which through 2 independent factors measures: the PA -in a first factor composed of 5 items-, assesses the existence of positive emotions and expressions: joy, pride, enthusiasm, energy, enjoyment (for example: “have you really felt happy?“), and the NA -in a second factor composed of 5 items-, assesses the existence of negative emotions and expressions: sadness, disgust, lethargy, fear, distress (for example: “have you felt unhappy or depressed?“); which is answered according to a scale of 4 points (1 little or nothing, 4 almost all the time). High scores on the PA scale show the predominance of positive emotions; while high scores on the NA scale show the predominance of negative emotions. In the present study the reliability index (omega) was 0.87 for PA and 0.85 for NA.

### Data analysis

All analyses were conducted using the R software [65]. Firstly, we assessed the assumptions of multivariate normality and homoscedasticity of the covariance matrices of the dependent variables using the Mardia test and Box’s M test, respectively. The results of the Mardia test provide evidence in favor of the multivariate normal distribution of the variables (skew = 377.11, *p* = .31; kurtosis=-0.62, *p* = .53). However, we did not obtain evidence in favor of the assumption of homogeneity of the variance-covariance matrices (*X*^*2*^_(78)_ = 150, *p* < .001). No outliers or missing cases were identified in the variables included in this study.

Subsequently, we conducted descriptive analyses (means, standard deviation, and bivariate correlations) for each construct as an observable variable (average of all indicators that compose it).

To test the hypothesis of our study, we performed a 2 × 3 multivariate analysis of covariance (MANCOVA [66]) using the Pillai’s trace test, which has been suggested when the assumption of homogeneity of variance-covariance matrices is not met [67], To assess significant results from MANCOVA, we conducted step-down covariance analyses (stepdown by Roy-Bargmann, [68]).

## Results

### Previous analyses

Table 2 presents descriptive statistics for divorce adjustment indicators. The results indicate that all pre-test measures are above the theoretical midpoint and below it for negative affect (i.e., 3 points in life satisfaction and 2.5 points for positive and negative affect). Regarding skewness and kurtosis, all variables are below |1.37| points. Bivariate correlations among divorce adjustment indicators are displayed in Table 3, showing moderate to high associations.

**Table 2:** Descriptive statistics of study variables (*N* = 30)

	M	SD	Me	min	max	Skewness	Kurtosis
Positive affect (pre-test)	2.85	0.77	2.90	1.20	4.00	-0.37	-0.86
Positive affect T1	2.75	0.67	2.70	1.40	4.00	0.13	-0.90
Positive affect T2	2.71	0.71	2.80	1.00	4.00	-0.55	-0.47
Positive affect T3	2.97	0.67	3.20	1.20	4.20	-0.54	-0.26
Negative affect (pre-test)	2.02	0.78	1.80	1.00	3.80	0.61	-0.68
Negative affect T1	2.10	0.73	2.00	1.00	3.40	0.22	-1.30
Negative affect T2	1.77	0.66	1.60	1.00	3.40	1.14	0.36
Negative affect T3	1.75	0.67	1.60	1.00	3.60	1.37	1.31
Life satisfaction (pre-test)	3.61	0.71	3.70	2.20	4.60	-0.31	-1.03
Life satisfaction T1	3.79	0.77	3.80	2.20	5.00	-0.21	-1.06
Life satisfaction T2	3.86	0.88	4.20	1.40	5.00	-0.83	-0.06
Life satisfaction T3	3.84	0.78	3.90	2.20	4.80	-0.49	-0.97

Note. ***M***: mean; ***Me***: median; **min**: minimum observed value; **max**: maximum observed value; **T1**: measurement at 3 months post-intervention; **T2**: measurement at 6 months post-intervention; **T3**: measurement at 12 months post-intervention.

**Table 3:** Pearson correlations among study variables (*N* = 30)

	1	2	3	4	5	6	7	8	9	10	11
1. PA Pre											
2. PA T1	0.63***										
3. PA T2	0.42*	0.68***									
4. PA T3	0.51**	0.56**	0.73***								
5. NA Pre	− 0.71***	− 0.65***	− 0.45*	− 0.32							
6. NA T1	− 0.55**	− 0.71***	− 0.49**	− 0.39*	0.73***						
7. NA T2	− 0.43*	− 0.63***	− 0.59**	− 0.38*	0.82***	0.67***					
8. NA T3	− 0.48**	− 0.65***	− 0.57**	− 0.53**	0.82***	0.65***	0.91***				
9. LS Pre	0.61***	0.62***	0.32	0.31	− 0.55**	− 0.69***	− 0.44*	− 0.42*			
10. LS T1	0.35	0.49**	0.04	0.06	− 0.49**	− 0.60***	− 0.46*	− 0.34	0.75***		
11. LS T2	0.02	0.24	0.05	0.09	− 0.05	− 0.20	− 0.14	− 0.03	0.43*	0.46*	
12. LS T3	0.02	0.45*	0.24	0.32	− 0.16	− 0.35	− 0.34	− 0.34	0.53**	0.50**	0.80***

Note. **Pre**: pre-test; **T1**: measurement at 3 months post-intervention; **T2**: measurement at 6 months post-intervention; **T3**: measurement at 12 months post-intervention; **PA**: Positive affect; **NA**: Negative affect; **LS**: Life satisfaction.

*p < .05; **p < .01; ***p < .001

### Hypothesis testing

To test the hypothesis of this study, we conducted a 2 × 3 mixed-design multivariate analysis of variance, considering pre-test measurements as covariates (MANCOVA). The intervention group was entered as the between-subjects factor (control and experimental groups), and repeated measures were treated as the within-subjects factor (time). The dependent variables were divorce adjustment indicators (i.e., negative affect, positive affect, and life satisfaction). Due to the violation of the sphericity assumption, we employed the Greenhouse-Geisser correction method. The analyses revealed significant results for the time factor (*p* < .001) and for the interaction between time and intervention group (*p* = .032). To evaluate the pattern of results obtained in the aforementioned interaction and assess if there were significant differences between groups (control and intervention), one-way MANCOVAs were conducted for each measurement time separately. No significant differences were found in any of them (*F*_(1,25)T1_= 1.19, *p* = .27; *F*_(1,25)T2_= 0.74, *p* = .40; *F*_(1,25)T3_= 0.25, *p* = .72). These results imply that there are no significant differences in divorce adjustment between groups at any measurement time. Subsequently, separate 2 × 3 mixed-design MANOVAs were conducted for each divorce adjustment indicator. Differences between groups were found in levels of reported negative affect over time (*F*_(1,25)_ = 1.62, *p* = .003). No significant differences were found for positive affects (*F*_(1,25)_ = 3.47, *p* = .07) or life satisfaction (*F*_(1,25)_ = 0.03, *p* = .86). Finally, to identify the measurement time at which differences between groups in negative affects occurred, step-down ANCOVAs (stepdown by Roy-Bargmann, [68]) were conducted with the intervention group entered as a dummy variable. First, we assessed the group effect on negative affects measured at three months post-intervention, controlling for pre-test effects. In the second model, we included the measurement of negative affects at six months post-intervention, controlling for pre-test effects and the three-month measurement. Finally, we included the measurement at twelve months post-intervention, controlling for pre-test effects and previous measurements (see Table 4). As shown in Table 4, no significant differences between groups were found at three months post-intervention (β= -0.19, SE = 0.14, *p* = .03). However, significant differences were observed at six months (β= -0.31, SE = 0.19, *p* = .32) and twelve months (β= -0.35, SE = 0.09, *p* < .001). In both cases, the level of negative affects was lower in the intervention group compared to the control group (*M*_*6months − control*_*=* 0.52, *M*_*6months − inter*_*=* 0.20; *M*_*12months − control*_*=* 0.38, *M*_*12months − inter*_*=* 0.03). These results suggest that the intervention had significant effects only in reducing adolescents’ negative affect, and this effect was evident at 6 months post-intervention and sustained over time.

**Table 4:** Analysis of covariance for negative affect

	Model 1 NA at 3 months			Model 2 NA at 6 months			Model 3 NA at 12 months		
Variables	Β	Es	*p*	Β	Es	*p*	Β	Es	*p*
Intercept	0.83	0.27	0.007	0.52	0.24	0.04	0.38	0.16	0.02
Intervention group	-0.19	0.19	0.32	**-0.31**	**0.14**	**0.03**	**-0.35**	**0.09**	**0.001**
Negative affect Pre-test	0.68	0.12	< 0.001	0.62	0.13	< 0.001	0.33	0.11	0.005
Negative affect 3 meses				0.07	0.14	0.60	-0.06	0.09	0.49
Negative affect 6 meses							0.56	0.12	< 0.001
Model Fit	*F*_(2,27)_ = 16.90, *p <* .001			*F*_(3,26)_ = 23.40, *p <* .001			*F*_(4,25)_ = 59.80, *p <* .001		

Note. **NA**: Negative affect

## Discussion

The aim of this study is to assess the impact of an attachment-based pilot intervention designed to enhance divorce adaptation in adolescents in terms of their subjective well-being. Through an experimental design, we evaluated changes in life satisfaction and positive and negative affect immediately after the intervention and then the maintenance of these changes in adolescents as a result of the intervention. The results partially support our hypothesis, as changes in the expected directions were observed in negative affect. However, no differences were identified in other dimensions of subjective well-being considered as indicators of divorce adaptation. The main findings of the study are discussed below.

Descriptive analyses reveal that before the intervention, adolescents reported moderately high levels of life satisfaction and positive affect, as well as predominantly low levels of negative affect. These results may suggest that despite being adolescents going through their parents’ divorce process, they do not experience noticeable difficulties in adapting to divorce. However, it is important to note that in a study with a Chilean sample comparing life satisfaction levels between adolescents from intact families and those whose parents had divorced, lower levels of life satisfaction were observed in the first group [9], in line with previous evidence (e.g., [7, 8]). Therefore, it could be inferred that even though adolescents whose parents are going through a divorce may report lower well-being indices, it does not imply that the consequences of divorce are particularly harmful to them. This could explain the relatively high levels of well-being reported by the evaluated sample before the intervention. At the same time, these findings challenge the notion that divorce is an experience associated with intense levels of distress, particularly considering that divorce is currently a highly frequent phenomenon and less socially sanctioned than decades ago. Taken together, these findings suggest that although adolescents may experience moderate levels of life satisfaction and positive emotions after their parents’ divorce, there is still room for interventions that enhance their adaptation process to this experience.

Regarding the main hypothesis of the study, which proposed that the intervention group would exhibit better levels of divorce adaptation (higher life satisfaction and positive affect, and lower negative affect) compared to those assigned to the control group, and that these changes would be maintained over time, only decreases in negative affect at 6 and 12 months after the intervention were observed. No significant differences between groups were found in individual measurements of life satisfaction and positive affect at any of the three measurement points. Specifically, the attachment-based intervention was effective in reducing negative affect in adolescents going through their parents’ separation process but did not increase life satisfaction or positive affect. While studies assessing the impact of attachment-based interventions to foster adaptation in adolescents from divorced parents are currently lacking, there is existing evidence supporting the effectiveness of interventions aimed at enhancing subjective well-being in both adolescents [69] and adults [70]. It is worth noting, however, that the effect size of these changes was relatively small, potentially complicating the identification of effects within a specific sample, such as the one evaluated in the present study.

A possible interpretation of these findings could be related to the temporal dynamics of emotional adaptation in adolescents to the divorce of their parents. At 3 months, it is plausible that participants may be immersed in an initial phase of emotional adjustment, where attachment-based intervention may not have had a significant impact. During this period, adolescents could be experiencing a range of intense emotions related to the transition, and consequently, the intervention may not have reached its full potential in terms of positively influencing negative affect. On the other hand, at 6 and 12 months, attachment-based intervention may have left a lasting impact on adolescents’ coping skills and perception of emotional support. It is plausible that, even though the intervention has concluded, the benefits continued to develop and manifest over time. As adolescents face long-term emotional challenges associated with parental divorce, the previously provided support could have translated into greater resilience and positive adaptation.

Thus, the intervention, focused on strengthening attachment bonds with parents, may have provided a secure space that allowed adolescents to acquire tools to manage unpleasant emotions and deploy more effective coping strategies, thereby dealing better with the challenges imposed by the family transition of divorce. The lack of effect on other dimensions of divorce adaptation can be explained from different perspectives. On one hand, it may be related to the possibility that the intervention was not sufficient to produce changes in the more stable aspects of well-being or in the overall perception of life, necessitating additional interventions, such as involving parents or extending the intervention duration. Cognitive subjective well-being (SWB) is commonly perceived as exhibiting greater long-term stability compared to affective SWB, which might be more susceptible to variations caused by everyday life occurrences [71]. On the other hand, it is also possible that the small sample size did not provide the necessary statistical power to detect effects if they were of small magnitude, especially considering that there was a (non-significant) trend toward an increase in levels of positive affect and life satisfaction. Therefore, further studies are needed to enhance this intervention or others aimed at promoting adjustment to divorce situations in this stage of the life cycle.

Specifically, our results support that a pilot intervention aimed at promoting a more secure attachment bond between adolescents and their parents resulted in a decrease in negative affect. This finding is consistent with previous research highlighting the protective role of a secure attachment bond and well-being [1, 33, 72].

The collective evidence reinforces the idea that, although adolescence is characterized by the pursuit of independence and autonomy, the context and family factors remain crucial for adolescents’ development [2, 29]. Therefore, promoting secure attachment bonds between parents and adolescent children, especially after divorce, is important for preventing exposure to risky situations that could harm their development. Similarly, having knowledge about the effectiveness of an intervention that works directly with adolescents opens a research avenue with potential implications for clinical practice. During adolescence, there is a transformation of hierarchical attachment relationships (typical of children), influenced by adolescents’ cognitive processes that allow them to question their primary attachment bonds. Therefore, this stage may be particularly crucial and receptive to the development of preventive interventions.

Although this study provides valuable information with potential clinical utility, it is not without limitations. The first limitation relates to the small sample size, which may have impacted the power to detect potential effects; for this reason, emphasis is placed on cautious interpretation of the data obtained in the study and its generalization. Therefore, the development of studies that replicate this intervention with a larger sample is recommended. Secondly, the strengthening of secure attachment (post-divorce) depends on both parents and adolescents, and a limitation of this study is not working with the dyad or triad. Future studies may explore the effects of interventions that consider a more comprehensive approach. Additionally, the potential influence of other stressful life events during the evaluated period should be controlled for in future studies. Lastly, no evidence was found for the assumption of homogeneity of matrices, which warrants a cautious interpretation of the results.

## Conclusion

The present study provides evidence that an attachment-based pilot intervention aimed at promoting adaptation to parental divorce in adolescents is capable of reducing negative affectivity over time. Future studies with larger samples could assess whether changes can be observed in other indicators of subjective well-being (life satisfaction and positive affectivity).

Our results emphasize the importance of fostering secure bonds between parents and adolescent children as a means of enhancing the well-being of adolescents facing the challenges posed by parental divorce.

## Data Availability

All materials (code and data) are available in a public repository hosted by the Open Science Framework https://osf.io/wvyrz/?view_only=78142df0ca4d4b529bcd42f8f59ad696.
